# Threat of Antimicrobial Resistance among Pilgrims with Infectious Diseases during Hajj: Lessons Learnt from COVID-19 Pandemic

**DOI:** 10.3390/antibiotics12081299

**Published:** 2023-08-08

**Authors:** Abdul Haseeb, Zikria Saleem, Hani Saleh Faidah, Abdullah A. Saati, Abdullmoin AlQarni, Muhammad Shahid Iqbal, Saleh Alghamdi, Mahmoud E. Elrggal, Manal AlGethamy, Rozan Mohammad Radwan, Ahmad Jamal Mahrous, Safa S. Almarzoky Abuhussain, Sarah M. Khayyat, Kiran Ibrahim, Brian Godman, Aziz Sheikh

**Affiliations:** 1 Department of Clinical Pharmacy, College of Pharmacy, Umm Al-Qura University, Makkah 24382, Saudi Arabia; 2 Department of Pharmacy Practice, Faculty of Pharmacy, Bahauddin Zakariya University, Multan 60800, Pakistan; 3 Department of Microbiology, Faculty of Medicine, Umm Al-Qura University, Makkah 24382, Saudi Arabia; 4 Department of Community Medicine & Pilgrims Healthcare, Faculty of Medicine, Umm Al-Qura University, Makkah 24382, Saudi Arabia; 5 Department of Infectious Diseases, Alnoor Specialist Hospital Makkah, Makkah 24382, Saudi Arabia; 6 Department of Clinical Pharmacy, College of Pharmacy, Prince Sattam Bin Abdulaziz University, Alkharj 11942, Saudi Arabia; 7 Department of Clinical Pharmacy, Faculty of Clinical Pharmacy, Al Baha University, Al Baha 57911, Saudi Arabia; 8 Department of Infection Prevention & Control Program, Alnoor Specialist Hospital Makkah, Makkah 24382, Saudi Arabia; 9 Pharmaceutical Care Department, Alnoor Specialist Hospital, Makkah 24382, Saudi Arabia; 10 Department of Epidemiology and Public Health, University of Nottingham, Nottingham NG7 2QL, UK; 11 School of Pharmacy, Sefako Makgatho Health Sciences University, Ga-Rankuwa, Pretoria 0208, South Africa; brian.godman@strath.ac.uk; 12 Strathclyde Institute of Pharmacy and Biomedical Sciences, Strathclyde University, Glasgow G4 0RE, UK; 13 Centre of Medical and Bio-Allied Health Sciences Research, Ajman University, Ajman 346, United Arab Emirates; 14 Usher Institute, The University of Edinburgh, Teviot Place, Edinburgh EH16 4UX, UK

**Keywords:** antimicrobial resistance, Hajj pilgrims, COVID-19, preventive measures, surveillance

## Abstract

Hajj pilgrimage is a large mass gathering global event that may facilitate the spread and emergence of various infectious diseases as well as antimicrobial resistance (AMR) in a local and global scenario. Planning and preparing for these public health issues is a challenging and complex process for the Kingdom of Saudi Arabia (KSA) health authorities. Despite multiple efforts for the prevention and treatment of infectious diseases through longtime funding in education and medical care, the prevalence of infectious disease is still high among Hajj pilgrims. The commonly observed infectious diseases during Hajj include respiratory tract infections (influenza and pneumonia), urinary tract infections and skin infections that may necessitate the use of antimicrobials. Beta-lactams are used as a first-line treatment for hospital acquired infections as well as community acquired infections due to their broad-spectrum activity. However, most of the bacterial isolates such as *Staphylococcus* spp., *Pseudomonas* spp. and *E. coli* are resistant to beta-lactams. Irrational use of antimicrobials, lack of infection prevention practices and suboptimal healthcare access further exacerbate the risk of spreading AMR among Hajj pilgrims. Enhanced collaboration between countries, sharing of best practices and international cooperation are crucial in addressing AMR threats among pilgrims. Consequently, robust surveillance systems for early detection and monitoring of AMR, collaboration with national as well as international healthcare agencies, effective infection prevention and control measures, public awareness and rational use of antimicrobials via antimicrobial stewardship programs are required to mitigate the risk of AMR and ensure the health and well-being of pilgrims during Hajj.

## 1. Introduction

An appreciable number of pilgrims gather from across the world to attend religious events and festivals when they occur [1]. Such gatherings increase the risk of especially communicable diseases that pose challenges to healthcare authorities [2,3,4]. Mass gatherings have been reported to have a considerable impact on an individual’s health and subsequent implications on the host’s country infrastructure and economy [5,6,7,8]. These mass gatherings can result in the dissemination of multi-drug resistant (MDR) strains and the spread of antimicrobial resistance (AMR) on a global scale [3,9,10,11]. Intersectoral approaches, public health surveillance and global communication are required to mitigate the risks of emerging and re-emerging infectious diseases [12,13,14,15,16]. After the H1N1 influenza pandemic, the first virtual international conference was held in Jeddah, KSA, in 2010, where The Lancet Infectious Diseases Series on mass gatherings was introduced. The majority of the national and international medical experts participated in this conference to develop and modify the guidelines for the diagnosis, treatment and prevention of infectious diseases during mass gathering events [17].

The Hajj is one of the biggest religious events hosted by the Kingdom of Saudi Arabia (KSA) every year. One of the obligatory pillars of Islam, Hajj is a pilgrimage to Makkah which is must for every physically fit, healthy Muslim to perform once in a lifetime if he/she can afford it [18]. It is performed from the 8th to 12th of Dhul-Hujjah, the last month of Islamic calendar. Currently, over three million Muslims travel to Saudi Arabia every year to perform Hajj rituals from almost 184 countries [19]. Over-crowded accommodations make an ideal environment for the exacerbation of communicable diseases, many of which are preventable if proper precautionary measures are taken [20]. Pilgrims are at potential risk of acquiring communicable diseases via contaminated food or water, person-to-person contact and vector-borne and respiratory transmission of viruses [4,17,21,22,23].

Antimicrobial resistant pathogens are currently prevalent in KSA [24,25,26]. Hajj pilgrims therefore face the potential risk of acquiring or transmitting these pathogens during their stay in KSA and subsequently spread these pathogens on their return home. A systematic review reported the prevalence and increasing trend of resistant pathogens during Hajj which include methicillin resistant *Staphylococcus aureus* (MRSA), 3GC-Enterobacteriaceae, colistin-resistant bacteria and imipenem-resistant bacteria [27]. Several studies conducted between 2002 and 2012 documented that the prevalence of MRSA varied significantly across different countries ranging from 0.06% to 94% [27,28,29].

The Hajj presents substantial logistic challenges for the protection of both non-residents and residents of KSA as well as the maintenance of local, national and international health security [17,30]. Infectious diseases including Zika virus diseases, extensive drug-resistant tuberculosis, (XDR TB), Middle East Respiratory Syndrome (MERS), seasonal influenza and severe acute respiratory syndrome coronavirus (SARS-CoV) have all surfaced in recent decades [31,32,33,34,35]. New policies by Saudi Ministry of Health have been implemented to try and reduce the transmission of infectious diseases during the Hajj. Policies include the administration of vaccines to pilgrims on arrival at KSA among unvaccinated pilgrims as well as decreasing the Hajj quotas for Saudi and non-Saudi pilgrims [36]. However, there is a need to build on current policies especially given concerns with increasing inappropriate antimicrobial use and resistance globally and the subsequent impact on morbidity, mortality and costs [37,38,39,40]. Consequently, we have summarized the available literature on infectious diseases and the use of antimicrobials among pilgrims. This is combined with recommended interventions to prevent possible emergence and transmission of local and global AMR. In addition, we highlighted the role of the Hajj pilgrimage in the spread of infectious diseases and AMR globally and the subsequent imperative to take necessary actions to address these urgent public health issues especially given the recent COVID-19 pandemic. Subsequently, we make recommendations for the future to key stakeholder groups in KSA and beyond based on the findings from the multiple studies included in this review.

## 2. Results and Discussion

### 2.1. Pattern and Prevalence of Infectious Diseases during Hajj

Possible infectious disease patterns during the Hajj pilgrimage include endemic, exported and imported diseases [41]. Inappropriate sanitary facilities, shared shelters, poor hygiene and lack of portable water enhance the transmission of infectious microorganisms. These combined factors have resulted in multiple communicable diseases among pilgrims during Hajj as well as after their return to their home countries [41,42]. The presence of a large number of pilgrims from different regions of the world in a gathering can increase the risk of spreading infectious diseases across international borders including resistant strains [43]. Climate conditions and air pollution in Makkah also play an important role in the transmission of infectious diseases [44].

Respiratory tract infections (RTIs) have been the predominant health problem among Hajj pilgrims over the past 15 years [45]. The most common pathogens for RTIs are *Klebsiella pneumoniae*, *Haemophilus influenzae*, Coronavirus, Adenovirus, respiratory syncytial virus (RSV), *Staphylococcus aureus* and *Streptococcus pneumoniae* [46,47]. According to one estimate, approximately 90% of Hajj pilgrims develop at least one respiratory illness before their return home [48]. Influenza has been the most common respiratory illness among Hajj pilgrims, estimated to be 24,000 cases per year [49]. Pneumonia has been observed as the most common life-threatening respiratory illness among pilgrims attending Mina healthcare centers [50] and the leading cause of hospital admissions particularly in intensive care units (ICUs) [51]. Among viral infections, herpes simplex virus (HSV) and adenovirus infections are the most commonly reported. The recent COVID-19 pandemic has also been a serious public health issue globally including in the KSA [52,53]. The KSA took all precautionary measures to prevent the spread COVID-19 based on typical effective public health measures and was declared COVID-19-free until March 2nd, 2020, when the first COVID-19 case was reported as an Iranian pilgrim [54,55]. Since then, KSA, the host country of annual Hajj pilgrimage, started witnessing an increasing trend of COVID-19 cases [56].

Moreover, of equal concern, is that tuberculosis (TB) has been reported in three studies [57,58,59]. The spread and emergence of MDR-TB has further complicated the circumstances, leading to unfavorable therapy outcomes and imposing an economic burden on patients as well as healthcare systems [57,60]. It is challenging to assess the exact prevalence of TB among Hajj pilgrims due to limited comprehensive studies targeting this specific group. Consequently, it is essential that the health authorities in KSA seek to implement strategies in the future to help control TB. These could include enhanced surveillance, diagnostics and treatment programs.

Several studies have, as mentioned, also recently discussed the transmission and acquisition of AMR during Hajj. Among the resistant strains, New Delhi metallo-B-lactamase, extended-spectrum B-lactamase-producing pathogens, SHV-12-producing *Salmonella typhi*, CTX-M-producing *Escherichia Coli*, *Streptococcus pneumoniae* [41] and methicillin-resistant *Staphylococcus aureus* (MRSA) have frequently been reported in Gulf Cooperation Council (GCC) countries especially in KSA [61,62]. The overall prevalence of infectious diseases among Hajj pilgrims is described in Table 1. Encouragingly, outbreaks of meningococcal disease during the Hajj have been largely prevented by a mandatory meningococcal vaccination policy for Hajj pilgrims, However, continued surveillance is needed to help prevent future outbreaks [59,63].

### 2.2. Patterns of Antimicrobial Use among Hajj Pilgrims

Pilgrims come from different regions of the world, including countries where antimicrobials are typically dispensed without a prescription, which contributes to the spread and emergence of resistance [84,85,86]. The purchasing of antimicrobials without a prescription is now less of an issue in KSA following tightening of the regulations and the potential for considerable fines for abuse [87]. One of the predominant factors in the dissemination of AMR among Hajj pilgrims is the irrational use of antimicrobials [11,41]. During the Hajj pilgrimage, both community-acquired and hospital-acquired infections may necessitate the use of antimicrobials. The selection of antimicrobials is determined by the specific type and severity of the infection. Beta-lactams and cephalosporins are commonly used antibiotics for hospital acquired infections which exhibit efficacy against a wide range of bacteria and are frequently utilized as first-line therapy [88]. However, glycopeptides, e.g., vancomycin, are increasingly being used for the treatment of serious infections caused by resistant pathogens such as MRSA [89]. Similarly, in the case of community-acquired infections, beta-lactams including amoxicillin are the most frequently prescribed antibiotics among outpatients [90]. Fluoroquinolones are also often prescribed for respiratory tract infections, including CAP as a first-line treatment [91]. Moreover, trimethoprim-sulfamethoxazole (TMP-SMX) and macrolides are also utilized for various infections such as respiratory, urinary and skin infections [34].

As reported in multiple studies, 34.9% of Australian pilgrims, 84% of Malaysian pilgrims, 17% of Pakistani pilgrims and 58.5% of Irani pilgrims received antimicrobials during Hajj [92,93,94,95] (Table 2). In another study, 47.6% of French pilgrims received antimicrobials [96] where beta-lactams (35.0%), macrolides (11.4%) and cephalosporins (2.3%) were the most common antimicrobials given to French pilgrims [96]. A prospective point prevalence study conducted in two referral hospitals in Medina documented that 49.2% of returning Hajj pilgrims were prescribed antibiotics. This included piperacillin-tazobactam (88%), penicillin (20%) and amoxiclav (12%) among Hajj pilgrims [97]. Another study reported that Malaysian pilgrims suffering from community-acquired pneumonia (CAP) acquired during the pilgrimage received levofloxacin (44%), azithromycin (40.7%) and cefuroxime (23.1%) on their return home [98].

**Figure 1:**
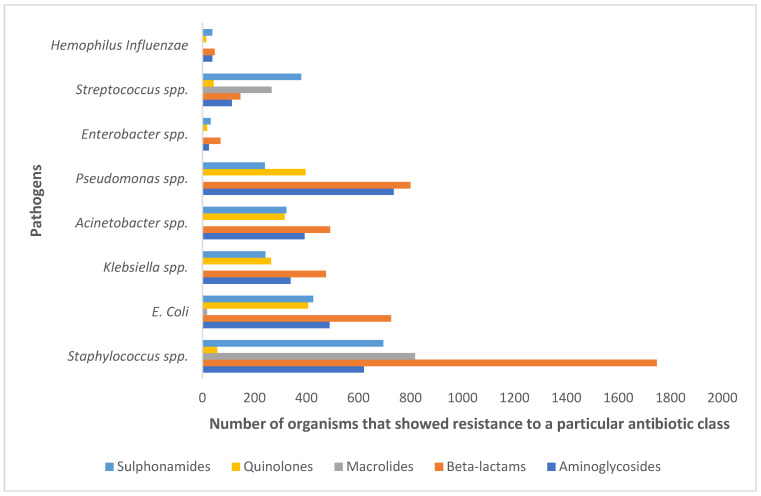
The most common AMR isolates reported during Hajj.

The most common AMR isolates reported during the Hajj season are illustrated in Figure 1, based on the findings of Alreeme et al. (2022) [99], with each bar representing the number of organisms resistant to a specific antibiotic class, e.g., macrolides, quinolones and beta-lactams. Among enteric disease-causing pathogens, *E. coli*, *Acinetobacter* spp., *klebsiella* spp., *Pseudomonas* spp. and *Enterobacter* spp. are the most common AMR isolates found in Hajj pilgrims while respiratory-disease causing AMR isolates include Staphylococcus spp., Streptococcus spp. and Hemophilus influenzae. Most of the pathogens are resistant to beta-lactams, followed by aminoglycosides and sulphonamides. Moreover, a study reported that Mycobacterium tuberculosis is resistant to streptomycin in 25.9% cases, while isoniazid showed resistance in 11.1% cases [57].

**Table 2:** Prevalence of antimicrobial use among Hajj pilgrims.

Author Year	Study Population (N)	Study Design	Prevalence of Antimicrobial Use	Top 3 Antimicrobials			Findings
				1	2	3	
Harimurti et al., 2021 [100]	Indonesian pilgrims (N = 813)	Prospective longitudinal study	47.8%	-	-	-	Pneumococcal vaccine should be administered before departure to KSA.
Alahmadi et al., 2020 [97]	Pilgrims from 7 different countries (N = 675)	Prospective point prevalence survey	49.18%	Penicillin (20%)	Amoxiclav (12%)	Pipercillin-tazobactum (88.0%)	The rational use of antimicrobial should be assessed by standardized methodology.
Hoang et al., 2019 [96]	French pilgrims (N = 783)	Prospective cohort study	47.6%	Beta-lactams (35.0%)	Macrolides (11.4%)	Cephalosporins (2.3%)	Educational training and sessions are required to control the irrational use of antimicrobials.
Alqahtoni et al., 2019 [101]	Pilgrims (N = 344)	Cross-sectional study	6%	-	-	-	Pre-travel education training related with health and use of preventive measure should be addressed.
Dzaralay et al., 2017 [98]	Malaysian Pilgrims (N = 91)	Cross-sectional study	100%	Levofloxacin (44%)	Azithromycin (40.7%)	Cefuroxime (23.1%)	The proper guidelines regarding antimicrobial use for the pilgrims with CAP should be introduced to improve healthcare services during Hajj.
Hashim et al., 2016 [70]	Malaysian pilgrims (N = 468)	Cross-sectional study	61.8%	-	-	-	Preventive measures including social distancing, wearing face mask, hand hygiene should be practice to prevent the spread of infectious diseases.
Metanat et al., 2015 [94]	Irani pilgrims (N = 422)	Prospective, cross-sectional study	58.5%	-	-	-	The meningococcal vaccine was effective in reducing the number of carriers among pilgrims after travel.
Azeem et al., 2014 [92]	Australian pilgrims (N = 229)	Cross-sectional study	34.9%	-	-	-	Educational sessions and campaign regarding rational use of antimicrobials is required.
Alborzi et al., 2008 [102]	Irani pilgrims (N = 674)	-	58.2%	-	-	-	The administration of vaccine was effective for reduction the number of carriers among pilgrims.
Mustafa et al., 2003 [93]	Malaysian pilgrims (N = 820)	Cohort study	84%	-	-	-	Immunization programs for Hajj pilgrims should be supported by KSA government
Qureshi et al., 2000 [95]	Pakistani pilgrims (N = 100)	Randomized blinded study	17%	-	-	-	Influenza vaccination should be recommended for the pilgrims before arrival to KSA.

KSA = Kingdom of Saudi Arabia.

### 2.3. Interventions and Recommendations

#### 2.3.1. Local and International Guidelines and Policies for Infection Prevention and Control

Infection prevention and control is a pivotal component of any healthcare system at a national as well international levels given rising rates of AMR and the implications [37,38,103]. Many infectious diseases as well as outbreaks are preventable if proper measures, including educational sessions regarding disease prevention and self-hygiene combined with prophylactic treatment including vaccines, are adopted by pilgrims before arrival to KSA [104]. Collaborative and well-coordinated efforts from all healthcare professionals (HCPs), as well as other key stakeholders and community groups, are needed to reduce future prevalence rates. To help with this, the KSA Ministry of Health provides up-to-date Hajj travel advice and health regulations through international public health organizations such as the Centers for Disease Control and Prevention (CDC), the WHO and Hajj travel agencies [20]. The WHO has published guidelines entitled “communicable disease alert and response for mass gathering” since June 2008 [105], with the recent COVID-19 pandemic focusing minds on key public health measures that can be introduced to stop the spread of infectious diseases. However, in view of continued concerns, it is recommended that Saudi Ministry of Health and public health officials should propose local guidelines for all stakeholders regarding infection prevention and control not for only future Hajj pilgrimages but also other mass gatherings. Table 3 depicts some recommended measures and their potential barriers that should be considered going forward to prevent or appreciably reduce infectious diseases during Hajj and their implications to both KSA and beyond.

#### 2.3.2. Restricting the Number of Hajj Pilgrims

The Saudi mitigation plan appears to have successfully limited the spread of COVID-19 in KSA as well as contributed to global health security [106]. In 2020, KSA authorities allowed 1000 pilgrims residing within KSA to perform Hajj with strict compliance with infection control measures and public health protocols [107]. No confirmed cases of COVID-19 or notable public heath events were recorded during this Hajj season. On the basis of the successful outcomes from the 2020 Hajj experience, Saudi authorities decided to extend the number to 60,000 pilgrims in 2021, presenting the similar results to 2020’s experience [108]. The rate of upper respiratory tract infections (URTIs) was 11.6 cases per 100,000 in the recent study compared to 2200 cases per 100,000 in a previous report [106]. Furthermore, a notable decrease in the number of non-communicable diseases (68 cases per 100,000) was reported when compared to previous study that showed the prevalence rate of 1600 per 100,000 cases [106,109]. The appreciable reduction in the cases of particularly URTIs reflects the effectiveness of adopting health policies and public health measures to restrict the number of Hajj pilgrims thereby ensuring their health to perform Hajj as well as reducing the period of Hajj stay alongside strict implementation of social distancing policies [106,110].

#### 2.3.3. Provision and Implementation of Adequate Healthcare Services

The KSA government provides over 1000 free healthcare facilities for all pilgrims during Hajj. The services include mass vaccination, outbreak investigation, environmental health services, infectious disease surveillance, mass administration of prophylactic medication and health education [30]. Interventions to cope with the dissemination of infectious diseases include non-pharmaceutical and pharmaceutical methods. Non-pharmaceutical methods include surveillance, wearing face masks, hand hygiene, social distancing, travel restrictions and respiratory etiquette, while pharmaceutical approaches include vaccination and the use of antimicrobials [111,112]. The strategies and policies should be introduced to improve vaccination coverage among all HCWs, and these strategies should be practiced by all healthcare facilities in Saudi Arabia [113].

##### Vaccination

Vaccination is the most effective way to prevent the acquisition and transmission of infectious diseases [114]. The WHO has estimated that approximately 2.5 million individuals are prevented from catching various infectious diseases through vaccination every year [115]. In addition, vaccines can not only protect individuals from serious disease but also unvaccinated individuals through the concept of herd immunity [116]. Moreover, several studies have supported the idea that administration of viral and bacterial vaccines help to control the emergence and spread of AMR [117,118,119]. Vaccine administration and acceptability can be promoted through the implementation of effective strategies including educating HCWs and pilgrims about vaccination as a prerequisite for acquiring a Hajj visa [19]. Such strategies are endorsed by the fact that the prevalence of influenza-like symptoms was lower in vaccinated pilgrims than in unvaccinated pilgrims [70].

In view of studies such as these, the Saudi Ministry of Health has recommended influenza and meningococcal vaccination as mandatory for all pilgrims entering KSA for the Hajj to reduce the risk of transmission of RTIs [120]. During the current COVID-19 pandemic, the Saudi healthcare authorities has also made COVID-19 vaccination a mandatory requirement for all pilgrims participating in Hajj rituals before leaving for KSA.

##### Hand Hygiene

Hand hygiene is one of the simple, primary and effective preventive measures recommended by various healthcare organizations for the prevention of cross-contamination of pilgrims especially during pandemics [121,122]. A survey of Australian and French pilgrims during 2013–2014 reported that 94% and 50% of their pilgrims, respectively, practiced various hand hygiene techniques including washing and sanitizing [19]. Generally, the use of alcoholic sanitizer is one of the essential hand hygiene practices to prevent infectious diseases. However, Muslim pilgrims are denied using them because alcohol is prohibited in Islam [123]. This is a concern as compliance with recommended hand hygiene was reported in US (67.2%) and Turkish (57%) pilgrims and was significantly associated with low risk of RTIs [124,125]. According to one study, the knowledge and attitude of Hajj pilgrims regarding the importance of hand hygiene was poor; however, compliance with hand washing was good [78]. Encouragingly, a recent study has reported a significantly lower incidence of RTIs among Hajj pilgrims during the COVID-19 pandemic after adopting hand hygiene practices [65].

##### Social Distancing and Contact Avoidance

According to the CDC, social distancing and contact avoidance with people are the best ways to minimize the transmission of infectious diseases [126]. During the COVID-19 pandemic, whilst no Hajj pilgrimage was performed in 2020 apart from 1000 KSA residents, in 2021 the Saudi healthcare authorities allowed the return of pilgrims. However, there were restrictions regarding social distancing of approximately 5 feet during prayers in the mosques and holy sites [127]. According to multiple surveys conducted among the wider pilgrim community, 48% of Turkish, 73% of Australian, 82% of Arab and 86% of French pilgrims believed that contact avoidance with sick people was a key element that would have reduced the transmission of infections [19].

##### Face Masks

Proper utilization of face masks has proven an effective preventive strategy to curb the aerosol spread of airborne infectious diseases. The effectiveness of face masks depends on its type, design and quality [128,129]. A study reported that Malaysian pilgrims used N-95 masks and surgical masks performing Hajj rituals [129]. However, the effectiveness of N-95 masks over surgical masks among HCWs from the prevention of communicable diseases is still unknown [130].

A meta-analysis study documented that the wearing face masks did not reduce the chances of catching influenza in 2009 [131]. Conversely, a systematic review reported that the prevalence of COVID-19, SARS and influenza decreased by 96%, 74% and 45% respectively by wearing facemasks [132]. In April 2020, the CDC recommended the use of cloth face masks to curtail community-based transmission [133], which should be adhered to for future mass gatherings.

### 2.4. Impact of Antibiotic Prescribing Patterns during the COVID-19 Pandemic on AMR

The irrational use of antibiotics during the recent COVID-19 pandemic may result in the emergence of AMR through appreciable over-prescribing across sectors despite limited evidence of bacterial infections or co-infections [11,134,135,136,137,138,139]. Usually, a large proportion of Hajj pilgrims consists of older people with multiple chronic comorbidities. Currently, patients with COVID-19 may receive antimicrobials for two main reasons. Firstly, the symptoms of the bacterial infectious disease resemble COVID-19. However, in order to differentiate between viral and bacterial infection, the ratio of CRP (mg/L) to 2–5A synthetase (pmole/dL) × 10 is used as a differential index. The index values in viral infections ranged from 0 to 0.9 and were lower than the values in bacterial infections, which ranged from 3.9 to 50 [140]. Diagnostic tests may though not be that effective with detection and can be time-consuming when immediate therapy is required [141]. Secondly, patients with COVID-19 may have bacterial co-infections that require antimicrobial therapy; however, this is rare in practice [137,138,139,142].

Consequently, comprehensive data are still required to have a better understanding of the occurrence of co-infections and pathogens involved, alongside the impact of underlying patient risk factors. Furthermore, standardized definitions and diagnostic criteria should be used to perform an in-depth analysis of microbiological resistance and antimicrobial usage where diagnostic laboratory infrastructure exists [143]. However, in the meantime, guidelines based on the AWaRe Book can be used to guide patient management of infectious diseases based on the balance of risks and benefits to reduce inappropriate prescribing and dispensing of antibiotics [144,145].

This is especially important in regions where Gram-negative pathogens are resistant to carbapenems. We are aware that antimicrobials with less favorable safety profiles such as colistin, a ‘Reserve’ antibiotic, are recommended as empiric therapy for suspected Gram-negative infections [146]. This needs to be avoided in the future. Similarly in countries such as Pakistan, ‘Watch’ and ‘Reserve’ antibiotics are being routinely dispensed in the community without a prescription driving up resistance rates [85], which is a concern. On the other hand, if antimicrobial treatment is not tailored to local AMR prevalence, patients with co-infections may receive ineffective therapy, which results in increased mortality rates and healthcare costs [142]. This situation can be avoided by developing local guidelines based on the AWaRe book and subsequently monitoring antibiotic usage through antimicrobial stewardship programs [147,148,149].

We are aware of a number of limitations with this study. Firstly, we restricted sourced papers to English language only for the reasons documented. Secondly, we utilized a limited number of databases including Scopus, PubMed and ScienceDirect, to retrieve articles; however, we believe these databases did capture most relevant papers of scientific interest. Thirdly, we did not assess the cause of irrational use of antibiotics that resulted in an increase in AMR as such studies have been conducted before. Despite these limitations, we believe our findings are robust providing direction to key stakeholders in KSA and beyond to reduce the extent of communicable diseases during Hajj

## 3. Materials and Methods

**Figure 2:**
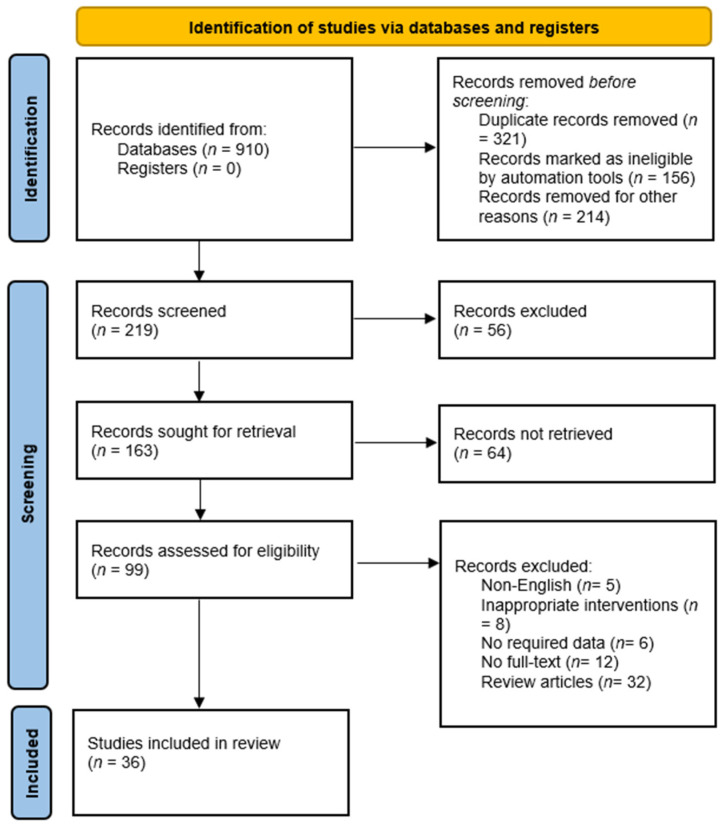
Flow chart of included studies.

This study is basically a narrative review of relevant published articles building on similar studies undertaken by some of the co-authors before in high priority areas including infectious diseases [148,150,151,152,153,154]. However, whilst there are no relevant formal reporting guidelines for narrative reviews, we built on guidance from the PRISMA-P (Preferred Reporting Items for Systematic review and Meta-Analysis Protocols) 2020 checklist PRISMA to enhance the structure and content of our paper (Figure 2) [155].

Initially, a scoping review was undertaken on Google Scholar to search available published literature and search keywords or key terms to structure a comprehensive search in potential databases. Table 4 contains the key terms used.

Subsequently, the identified key terms were entered in 3 databases. These were Google Scholar, PubMed and ScienceDirect to search for relevant articles. We were only interested in publications published in English as this is the internationally accepted scientific language. Potential publications were searched from January 2000 until September 2022 to provide the most recent data as concerns with the transmission of infectious diseases have grown following the COVID-19 pandemic alongside fears with increasing AMR and the implications. Potential studies for inclusion in the review were divided among the co-authors using specifically designed data collection forms. The findings from identified papers were accumulated and assembled, and the results were summarized in the form of tables and descriptions by the principal author (ZS). This approach, as mentioned, aligns with similar studies undertaken by the co-authors across Africa and beyond, in giving future guidance for managing infectious diseases and broader approaches and in accordance with institutional guidance [16,148,150,151,152,153,154].

## 4. Conclusions

Hajj pilgrimage contributes towards the transmission of communicable diseases around the globe. This narrative review highlights the importance of intersectoral government collaboration for educational sessions regarding the rational use of antimicrobials before and during Hajj pilgrimage. This is essential given rising concerns with AMR globally. Alongside this, the need for vaccination against target viruses among pilgrims including COVID-19, influenza and meningococcus, as well as acknowledging the need for personal hygiene (face masks and hand hygiene) and social distancing during Hajj to minimize the risk of transmission of various infectious diseases and the risk of AMR is essential. This requires co-ordination among key stakeholder groups in KSA, including educational activities, as well as international collaboration and strategies. These include expanded AMR surveillance, increased laboratory diagnostic testing and infection prevention and control programs, in addition to educational and other programs to address vaccine hesitancy where this occurs. These coordinated efforts are required to overcome this global public health concern and ensure Hajj pilgrims return home safely without any infectious diseases. These are research projects for the future.

## Figures and Tables

**Table 1:** Prevalence of infectious diseases among Hajj pilgrims.

Author Year	Geographical Origin	Study Design	Sample Size	Prevalence of Infection	Top 3 Infections			Findings
					1	2	3	
Mahdi et al., 2022 [64]	Makkah	Cross-sectional study	476	2.3%	RTIs (2.3%)	-	-	Low prevalence of RTIs among pilgrims was observed as compared to those documented in pre-pandemic studies.
Mahdi et al., 2022 [65]	Makkah	Cohort study	445	4.7%	RTIs (4.7%)	-	-	Hand hygiene practice could play an important role to reduce the prevalence of RTIs.
Alasmari et al., 2021 [66]	Jeddah	Cross-sectional study	2973	4.6%	Neisseria meningitidis (4.6%)	-	-	Vaccination is required to prevent meningococcal disease outbreaks during and after Hajj.
Al-Hayani et al., 2021 [58]	Makkah	Cross-sectional study	472	100%	Pulmonary tuberculosis (91.7%)	-	-	Epidemiological studies are needed to validate the findings.
Sambas et al., 2020 [57]	Makkah	Cross-sectional study	158	100%	Tuberculosis (100%)	-	-	TB control programs are required to prevent emergence and spread of MDR TB.
AboEl-Magd et al., 2020 [67]	Makkah	Cohort study	614	39.2%	Pneumonia (39.2%)	-	-	Upgradation of antibiograms is required to enable selection of appropriate antibiotic.
Raja et al., 2017 [68]	Makkah, Madina, Jeddah	Descriptive, cross-sectional study	184496	30.0%	RTIs (29%)	-	-	Structured policies and legislation, training sessions of HCWs should be introduced to prevent the spread of infections.
Yezli et al., 2017 [59]	Makkah	Cross-sectional study	1063	1.4%	Tuberculosis (1.4%)	-	-	Undiagnosed TB poses a risk to other pilgrims. Consequently, proactive screening is needed.
Shirah et al., 2017 [43]	Madina	Retrospective study	1059	23%	Pneumonia (23%)	-	-	Specific adjustment in the guidelines is required for the treatment of pneumonia.
Dhafar et al., 2016 [69]	Makkah	Descriptive, observational study	217	-	RTIs (12.9%)	Skin and soft tissue infections (9.2%)	-	Policies and legislation are required to improve the quality life of patients.
Hashim et al., 2016 [70]	Makkah, Arafat	Cross-sectional study	468	93.4%	-	-	-	Preventive measures should be practiced.
Bakhsh et al., 2015 [71]	Makkah	Observational study	1008	-	RTIs (17.6%)	Skin and soft tissues infections (15.7%)	-	Improvement in healthcare facilities during Hajj is required.
Memish et al., 2014 [72]	Makkah and Madina	Observational study	38	68.4%	CAP (68.4%)	-	-	Improved respiratory tract infection surveillance is needed.
Memish et al., 2013 [73]	Makkah and Madina	Observational study	1103	100%	Invasive meningococcal disease (100%)	-	-	The number of cases has declined. Regular monitoring is necessary to monitor the trends during upcoming hajj seasons.
Barasheed et al., 2014 [74]	Makkah, Mina	Randomized controlled trial	1038	38%	Rhinovirus (25%)	Influenza (2%)	Adenovirus (2%)	Appropriate vaccination and infection control are required to reduce the risk of transmission of respiratory virus.
Alzahrani et., 2012 [75]	Mina	Descriptive study	4136	67%	RTIs (60.8%)	Infectious skin diseases (4.7%)	UTIs (1.5%)	Best possible healthcare service should be given to Hajj pilgrims by Saudi healthcare authorities.
Mandourah et., 2012 [76]	Mina, Makkah, Arafat, Madina	Cohort study	452	27.2%	CAP (66.7%)	Aspiration-related pneumonia (25.2%)	Tuberculosis (4.9%)	Increased efforts for the prevention of infectious diseases among Hajj pilgrim is required.
Alherabi et al., 2011 [77]	Makkah	Cross-sectional study	3087	92%	Pharyngitis (45.7%)	URTIs (42.1%)	Influenza (2.5%)	Misuse of antimicrobials should be discouraged by guiding pilgrims regarding rational use of antimicrobials.
Al-Ghamdi et al., 2011 [78]	Mina, Arafat	Cohort study	160	57%	Pneumonia (39.4%)	URTIs (3.3%)	-	Structured policies and strategies regarding infection prevention and control should be initiated.
Baharoon et al., 2009 [79]	Makkah	Cross-sectional study	165	71%	CAP (54.8%)	Intra-abdominal source (16.6%)	Skin and soft issue infection (14.3%)	Initiation and implementation of infection prevention and control programs are required.
Ibrahim et al., 2008 [80]	Mina	Cross-sectional study	248	-	RTIs (29.8%)	UTIS (1.6%)	-	Intensified health education campaigns should be conducted for all pilgrims in their home countries and KSA.
Madani et al., 2007 [81]	Mina, Arafat	Cross-sectional Study	140	26.4%	Pneumonia (22%)	Sepsis (4.3%)	-	Cost-effective and optimal healthcare services are urgently needed for Hajj pilgrims.
Madani et al., 2006 [82]	Mina, Arafat	Cross-sectional study	808	36.4%	Pneumonia (19.7%)	URTIs (3.3%)	Cellulitis (1.6%)	Cost-effective and optimal healthcare services are urgently needed for Hajj pilgrims.
Memish et al., 2006 [61]	Mina	Cohort study	411	20.6%	-	-	-	Susceptibility testing should be performed so that antimicrobials could be used when needed.
Balkhy et al., 2004 [49]	Mina	Cross-sectional study	500	10.8%	Influenza (55.6%)	HSV (24.1%)	RSV (12.9%)	Vaccination should be required for every Hajj pilgrim.
Karima et al., 2003 [83]	Makkah	Cross-sectional study	105	100%	Meningitis (64%)	Meningococcemia (36%)	-	Quadrivalent Vaccine is required for all pilgrims before coming to KSA.

RTIs; Respiratory tract infections, UTIs; Urinary tract infections, CAP; Community-acquired pneumonia, HSV; Herpes simplex virus.

**Table 3:** Recommended strategies and their potential barriers to prevent infection during Hajj.

Strategies	Potential Barriers
Public Awareness	Language barriers. Illiteracy. Scarcity of resources for educational programs. Concerns with misinformation from authorities
Adequate sanitation facilities	Limited access to clean water and sanitation facilities. Overcrowding. Insufficient availability of handwashing stations. Lack of awareness of hand hygiene.
Respiratory Etiquettes	Culture norms. Lack of awareness about respiratory hygiene practices.
Vaccination campaign	Limited access to vaccines. Vaccines hesitancy. Inadequate healthcare infrastructure.
Infectious Disease surveillance	Lack of resources for surveillance. Delays in reporting and response.
Crowd management and planning	Lack of infrastructure for crowd control. Logistical challenges.
Food Safety Measures	Poor food handling practices. Lack of proper food inspection and regulation.
Healthcare Services availability	Insufficient healthcare facilities and personnels. Overwhelmed healthcare systems.

**Table 4:** Retrieval strategy and search results from Databases.

#	Search Terms
1	(Hajj) OR (Pilgrims)-{MeSH Terms} OR/AND {Text Word}
2	(Antimicrobial resistance) OR (Antimicrobial sensitivity) - {MeSH Terms} OR/AND {Text Word}
3	(Infectious diseases) AND (Antimicrobial use)-{MeSH Terms} OR/AND {Text Word}
4	(COVID)-{MeSH Terms} OR/AND {Text Word}
5	#1 AND #2
6	#1 AND #3
8	#2 AND #3
9	#1 AND #2 AND #3 AND #4

## Data Availability

No data are available. All data relevant to the study are included in the article or uploaded as supplemental information.
